# Supplementary material for: Genetic Dissection of CRISPR-Cas9 Mediated Inheritance of Independently Targeted Alleles in Tobacco α-1,3-Fucosyltransferase 1 and β-1,2-Xylosyltransferase 1 Loci
Source: Int J Mol Sci. 2022 Feb 23;23(5):2450. doi: 10.3390/ijms23052450 (PMC8910323; doi:10.3390/ijms23052450)
Supplement: Supplementary file 1 [file ijms-23-02450-s001.zip › Table S1.pdf]

Table S1. gRNA properties used in the experiment.

Target gene	Vector	sgRNA				
		name	GC content	Out-of-frame score	Mismatches	Direction
NbFucT1	pGenovo111	sgRNAFucT1	30.00%	59.90%	0	-
NbXylT1		sgRNAXylT1	60.00%	60.50%	0	-
