# Supplementary material for: Genetic Dissection of CRISPR-Cas9 Mediated Inheritance of Independently Targeted Alleles in Tobacco α-1,3-Fucosyltransferase 1 and β-1,2-Xylosyltransferase 1 Loci
Source: Int J Mol Sci. 2022 Feb 23;23(5):2450. doi: 10.3390/ijms23052450 (PMC8910323; doi:10.3390/ijms23052450)
Supplement: Supplementary file 1 [file ijms-23-02450-s001.zip › Table S2.pdf]

Table S2. Primer information used in this study.

Use	Primer name	Primer sequence information (5'-3')
For Sanger sequencing	NbFucT1-F	GTCAAATTCAAACGCACCCA
	NbFucT1-R	ATCCCTCGAATAAGCCACAG
	NbXylT1-F	GAACGTTGTTTAACCCTCTG
	NbXylT1-R	ATATCGTCCGGATTCATTCG
For targeted deep sequencing	NbFucT1-1st-F	ATGAGATCGGCGTCAAATTCAAACG
	NbFucT1-1st-R	ATCCCTCGAATAAGCCACAGAATC
	NbFucT1-2nd-F	ACACTCTTTCCCTACACGACGCTCTTCCGATCTCAAACGCACCCAATAAGCA
	NbFucT1-2nd-R	GTGACTGGAGTTCAGACGTGTGCTCTTCCGATCTTGATAAAACGATTCGGC
	NbXylT1-1st-F	GAACGTTGTTTAACCCTCTGTAGTC
	NbXylT1-1st-R	ATATCGTCCGGATTCATTCGTATTGC
	NbXylT1-2nd-F	ACACTCTTTCCCTACACGACGCTCTTCCGATCTTCGCTCTCAACTCAATC
	NbXylT1-2nd-R	GTGACTGGAGTTCAGACGTGTGCTCTTCCGATCTCTTGGAATATTGAG
Cas9 detection	Cas9-F	CCCACCATCTACCATCTGCG
	Cas9-R	ATGTCCTCGTTCTCCTCGTTGT
