# Supplementary figures and images for: Genetic Dissection of CRISPR-Cas9 Mediated Inheritance of Independently Targeted Alleles in Tobacco α-1,3-Fucosyltransferase 1 and β-1,2-Xylosyltransferase 1 Loci
Source: Int J Mol Sci. 2022 Feb 23;23(5):2450. doi: 10.3390/ijms23052450 (PMC8910323; doi:10.3390/ijms23052450)

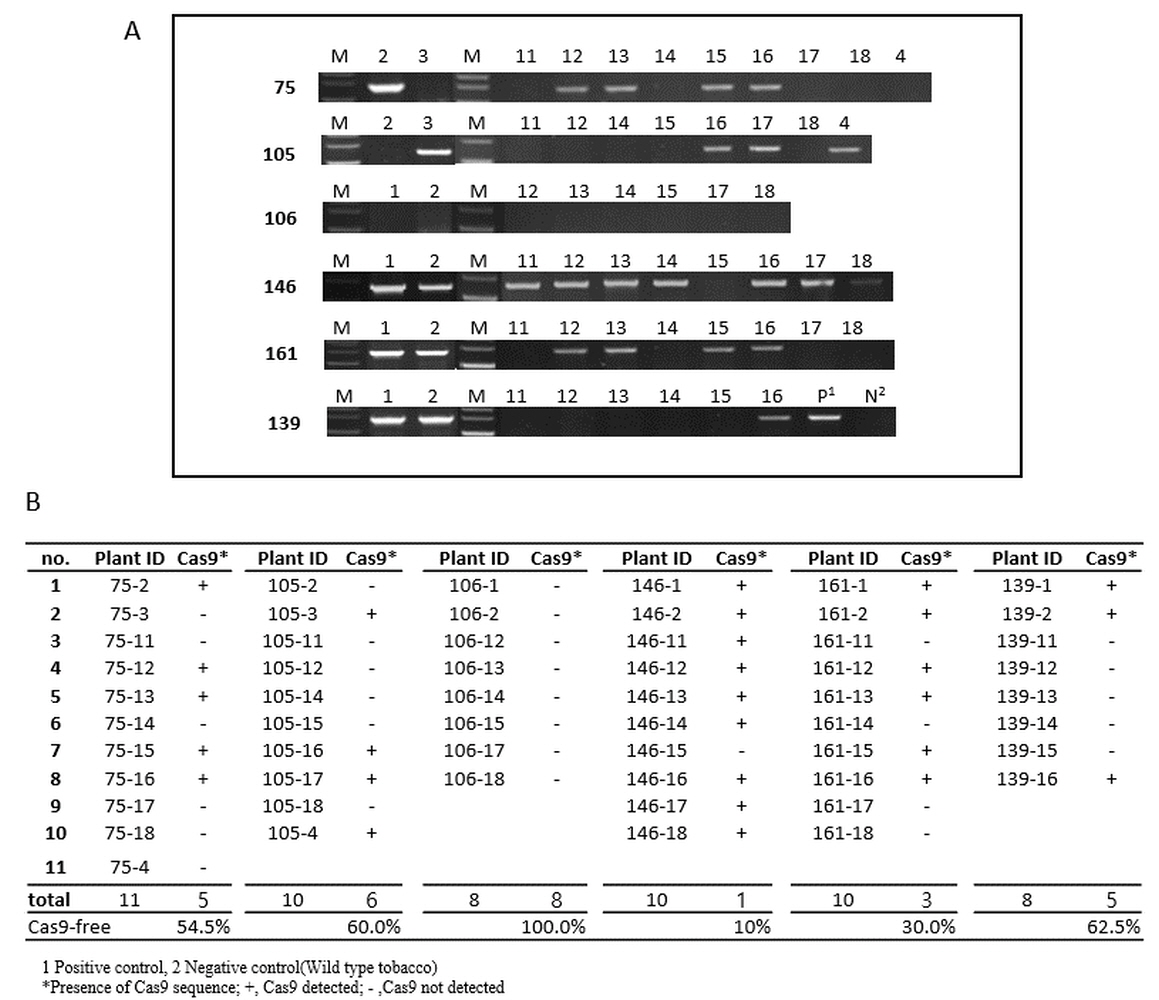

Supplement: Supplementary file 1 [file ijms-23-02450-s001.zip › Figure S1.jpg]
